# Bidirectional analysis of clinical and MRI correlations in temporomandibular disorders using regression models

**DOI:** 10.1007/s11282-025-00894-3

**Published:** 2026-01-19

**Authors:** Péter Schmidt, Szilvia Ambrus, Szandra Körmendi, Máté Jász, Mihály Vaszilkó, Bálint Jász, Bence Tamás Szabó, Adrienn Dobai

**Affiliations:** 1 Department of Prosthodontics, Faculty of Dentistry, Semmelweis University, Szentkirályi u. 47, Budapest, 1088 Hungary; 2 Department of Oro-Maxillofacial Surgery and Stomatology, Semmelweis University, Mária u. 52, Budapest, 1085 Hungary; 3 Department of Oral Diagnostics, Faculty of Dentistry, Semmelweis University, Szentkirályi u. 47, Budapest, 1088 Hungary

**Keywords:** Diagnostic criteria for temporomandibular disorders, MRI, Temporomandibular joint

## Abstract

**Objectives:**

In the course of a standard diagnostic procedure for temporomandibular joint disorder (TMD), there is often uncertainty regarding the necessity of magnetic resonance imaging (MRI) of the temporomandibular joint (TMJ). This study aims to clarify the relationship between clinical TMD symptoms and MRI findings using logistic regression models, to better define the role of MRI in diagnostics.

**Methods:**

In this retrospective study, the authors analysed a sample of 80 temporomandibular joints (TMJs). Forty patients with TMD symptoms were selected for the study, all of which had previously undergone examination in accordance with the diagnostic criteria for temporomandibular disorders (DC/TMD), as well as having undergone TMJ MRI. Descriptive statistics and regression analyses were used to explore any correlation between clinical symptoms and MRI findings.

**Results:**

MRI-based explanation of clinical symptoms revealed thirteen significant regression models with the following dependent variables: palpation pain at the lateral TMJ pole, TMJ crepitation, condylar hypermobility, uncorrected mandibular deviation, and palpation pain in the medial and lateral pterygoid muscles, as well as in the masseter and temporalis muscles. In contrast, the clinical symptom-based inference of MRI diagnoses yielded eleven significant models, with MRI findings as dependent variables: effusion, degenerative joint disease, anterior disc displacement without reduction, medial disc displacement, thickening at the insertion of the lateral pterygoid muscle, subluxation of the mandibular condyle, reduced glenoid fossa height, and abnormal disc morphology. Among all models, only anterior disc displacement without reduction with condylar hypermobility and with the pain in the masseter muscle demonstrated acceptable predictive accuracy. (AUC = 0.651, AUC = 0.637).

**Conclusions:**

This study confirms that clinical examination alone may be insufficient for accurately diagnosing specific TMJ pathologies. Although some clinical signs show strong associations with MRI findings, only two regression models demonstrated acceptable predictive value.

## Introduction

Temporomandibular disorders (TMD) are multi-etiological conditions of the temporomandibular joint (TMJ), the masticatory muscles, and the associated structures presenting the same main symptoms: pain in the orofacial region, joint sounds, or limited range of mandibular movement. TMD nomenclature and clinical assessment were standardized by the Research Diagnostic Criteria for Temporomandibular Disorders (RDC/TMD), and more recently its successor, the Diagnostic Criteria for Temporomandibular Disorders (DC/TMD) [1–3]. The articular disc, the muscles and the bony components of the TMJ can be evaluated by magnetic resonance imaging (MRI) [4]. 

In the course of the standard diagnostic procedure for TMD (Temporomandibular Joint Dysfunction), there is frequently a lack of clarity regarding the appropriateness of utilising imaging procedures. The position statement of the American Academy of Oral and Maxillofacial Radiology and the American Academy of Orofacial Pain asserts that magnetic resonance imaging (MRI) is recommended for the evaluation of suspected disc displacement [5]. According to the DC/TMD, the diagnostic criteria for a common TMD subgroup, disc displacement with reduction (DDwR), are the presence of opening and/or closing sound during clinical examination, in addition to the manifestation of this clicking sound in the preceding thirty days, as reported by the patient. In order to confirm a diagnosis of this internal derangement, the gold standard is an MRI recording of the jaw in closed and open positions. The recommendation to evaluate or confirm the disc position is consistent for all intracapsular derangements [6]. The MRI of the temporomandibular joint has been shown to facilitate the detection of additional information that is of clinical relevance. This includes the presence of early signs of joint effusion, as well as morphological changes to structures in question [7]. 

The relationship between the clinical manifestations of TMD and the anatomical and pathological findings of the temporomandibular joint on magnetic resonance images were previously investigated using a variety of statistical methods [8–19]. However, the majority of these methods did not incorporate regression models. Furthermore, the MRI signs were examined as pathologies behind the clinical symptoms, but only three studies investigated what changes we can expect on MRI in the presence of certain clinical symptoms [17–19]. 

The aim of the present study was to determine which pathologies, as seen on MRI, could be responsible for the different clinical symptoms, and which clinical symptoms could be used to predict the different MRI signs, thus improving the MRI diagnosis. A range of regression analyses were performed between the clinical symptoms and the MRI findings in order to examine their relationships.

## Materials and methods

### Patients

In this study, the records of forty patients from the Department of Prosthodontics at Semmelweis University were retrospectively analysed, all of which had visited the department with at least one temporomandibular disorders symptom (pain, limited range of movements, clicking sound, and/or crepitation) between October 2022 and March 2023. As an inclusion criterion, all patients had previously undergone examination based on the Diagnostic Criteria of Temporomandibular Disorders (DC/TMD) following a comprehensive anamnesis, and they had also undergone TMJ MRI examination. Additional selection criteria required that all patients had been managed by the same clinician and that all MR images be evaluated by a single radiologist. This approach was chosen to ensure consistent application of the assessment protocol throughout the study. The following exclusion criteria were applied: (1) Age under 18 years. (2) Previous orthognathic surgery or orthodontic treatment. (3) Presence of relevant craniofacial deformity. (4) Previously confirmed TMJ fractures or systemic diseases, such as rheumatoid arthritis. (5) Inaccurate and/or missing documentation during the physical examination. (6) More than two weeks between the clinical and radiological examinations. (7) Therapeutic intervention between the clinical examination and the MR imaging. (8) Previously confirmed autoimmune and connective tissue disease. Consequently, all the selected patients had a fully completed DC/TMD symptom questionnaire. This retrospective study was conducted in accordance with the medical protocols and ethical principles outlined in the Declaration of Helsinki. In light of the retrospective nature of this study, ethical approval of the study was waived by the institutional review board of Semmelweis University (214/2023).

### Clinical examination

The clinical examinations of the temporomandibular joints, masticatory muscles, and the surrounding area were performed according to the Diagnostic Criteria for Temporomandibular Disorders (DC/TMD), and data were collected by a dentist with 20 years of clinical experience in the field of TMD (P.S.). Cases with missing data were excluded from the study. The results obtained from the clinical findings presented in this research are outlined in Table 1. Pain in the temporomandibular joint and masticatory muscles was registered based on the patient’s pain experience in the last 30 days and on palpation findings. The presence of muscle pain (due to a pressure of 1 kg and 0.5 kg according to the DC/TMD examination protocol, determined by Wagner Paintest FPX 25 algometer, Wagner Instruments, USA) was investigated in the temporal and masseter muscles, in the lateral pterygoid area, and in two additional sites: the medial pterygoid muscle and the sternocleidomastoid muscle. In addition, ear symptoms (pain, seizure, popping) were recorded. Range of movements (pain-free, maximum active and passive opening, left and right lateral and protrusive movements) and presence of clicking sound(s) and crepitation of the temporomandibular joint during opening, closing, and horizontal movements were registered. TMJ hypermobility, i.e. clicks at the end of wide opening and at the beginning of closing were also registered additionally [20]. Limited mouth opening was defined in accordance with the patients complains.

### Magnetic resonance imaging of the TMJ

**Fig. 1:**
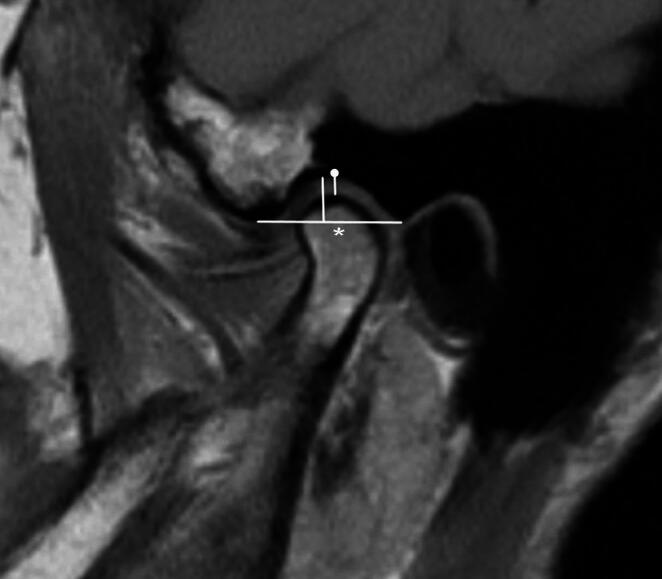
Height and anteroposterior diameter of the glenoid fossa and width of the TMJ space. The white circle illustrates the uppermost point of the glenoid fossa; the white star shows the centre of the mandibular condyle

A 1.5 T imager (Signa Voyager 1.5T; General Electric) with a head coil was used for imaging the TMJ in all subjects. The imaging protocol included PD-weighted, T1-weighted, T2-weighted, and T2-weighted fat-saturated image sequences. A sagittal scan was set to be parallel to the short axis of the condylar head, and a coronal scan was set to be parallel to the long axis of the mandibular condyle. As all TMJ MRI examinations were made on the same machine, the parameters used for TMJ imaging were consistent for each patient: PD images; TR 1889 ms, TE 12 ms, and T2 weighted images with fat saturation; TR 2300 ms, TE 83 ms, T1 weighted images; TR 634 ms, TE 15 ms, FOV 140 × 126 mm, slice thickness 2 mm and slice intervals 0.2 mm. MRI was taken in occlusion and in maximum mouth opening position. In order to maintain this open position, bite wedges with millimetre sets were used and always adjusted to the maximum mouth opening size registered and communicated by the clinical examiner. Each MRI examination was conducted within one week after the clinical assessment. The radiologic report was written by the same dentomaxillofacial and head and neck radiologist. As these reports originally did not contain morphometric parameters of the glenoid fossa such as height and width, the same radiologist who had written the reports, measured these morphometric factors retrospectively. The MRI signs analysed in this research were the following:

- **Effusion**: This study involved the observation of effusion with higher than 2 mm thickening, which was reported as marked or extensive effusion in the radiological report.
- **Bone marrow edema**: Bone marrow edema in the condylar head was indicated by a hypointense signal on T1- and hyperintense signal on T2-weighted fat saturated images.
- **Degenerative joint disease (DJD)**: Presence of obvious erosion, flattening, sclerosis, and osteophyte was documented as pathological.
- **Internal derangements**: Anterior DDwR, anterior disc displacement without reduction (DDwoR), posterior, medial, and lateral disc displacements were detected. Normal disc position was defined by the location of the posterior band of the disc at the superior or 12 o’clock position relative to the condyle [21].
- **Abnormal disc morphology**: Flattened, folded, and convex disc configuration was considered as an abnormal disc. A biconcave form was designated as normal. All disc configurations were evaluated in the closed mouth position.
- **Lateral pterygoid muscle insertion thickening**: Thickened area with low signal intensity in the insertion of the lateral pterygoid muscle. This sign is typically associated with an anterior dislocation of the disc, and the radiological report has described this thickening as a pterygoid double disc sign.
- **Mandibular condyle subluxation**: The mandibular condyle moves over the articular eminence in maximal opening position.
- **Quantitative morphological measurements**: The anteroposterior diameter of the glenoid fossa, its height, and the width of the TMJ space were measured on a standardized sagittal plane. (Fig. 1.) Glenoid fossa was grouped as follows according to height: ≤ 6 mm and > 6 mm. TMJ space was divided into two groups according to width: normal ≥ 3 mm, narrow < 3 mm.

#### Mandibular condyle position:

Anterior, normal, and posterior positions were categorised based on the condyle centre position in relation to the uppermost point of the glenoid fossa. (Fig. 1).

### Statistical analysis

Prior sample size estimation was performed using G*Power software (version 3.1; Universität Düsseldorf, Germany), targeting a statistical power of 80% and a significance level of 0.05. Based on these parameters and the expected effect size, the required sample size was determined to be 77 temporomandibular joints (TMJs). Descriptive statistics were used to analyse the frequencies of clinical and MRI findings. Associations between clinical and radiological signs were examined in two ways to assess the following:

- Regression models analysing clinical signs as dependent variables, to reveal MRI abnormalities behind different clinical signs.
- Regression models analysing MRI signs as dependent variables to investigate how MRI diagnosis can be predicted based on clinical examination.

To answer these questions logistic regression models were used. The fitted model was selected based on the P-value at a significance level of 0.05. In models examining the clinical signs, the following MRI pathologies were used as independent variables: effusion, degenerative joint disease, anteriorly displaced disc with reduction, anteriorly displaced disc without reduction, posteriorly displaced disc, medially displaced disc, laterally displaced disc, abnormal disc morphology, lateral pterygoid muscle insertion thickening, mandibular condyle subluxation, TMJ space width, glenoid fossa height and width and posterior position of mandibular condyle. In the models, the independent variables forecasting the MRI diagnosis were the following clinical symptoms: clicking, crepitation, condylar hypermobility, reciprocal click, limited mouth opening, corrected and uncorrected deviation, pain of the lateral TMJ pole, palpation pain in the masseter muscle, palpation pain in the medial pterygoid muscle, palpation pain in the lateral pterygoid muscle and palpation pain in the temporal muscle. Other parameters, e.g. patient’s reports were not included in these models to make them as reliable as possible and reduce subjectivity.

In the model to determine the relevance of the presence of certain clinical signs for the prognosing the MRI diagnosis, a ROC curve was used. In general, an area under the curve AUC of 0.5 suggests no discrimination, 0.7 to 0.8 is considered acceptable, 0.8 to 0.9 is considered excellent, and more than 0.9 is considered outstanding [22]. 

## Results

### Descriptive analysis

Twenty-six females and fourteen males were selected with a mean age of 42 (SD ± 16.19). Tables 1. and 2. present the frequency and descriptive analysis of the clinical and radiological findings.

TMD pain was reported by 55% of the patients, while the clinician registered palpation pain between 10% and 63.7% depending on the examined region. Of the 80 temporomandibular joints examined, a total of 41 were detected with clicking and 11 with crepitation, while 70% of the patients reported TMJ sounds. Condylar hypermobility was found in 41.3% of the joints, while 46.3% of TMJ’s showed a reciprocal click. Ear symptoms (pain, popping, seizures) were present in 23 out of the 80 examined sites. Only 5% of the patients had limited mouth opening, and 32.5% and 45% of the patients had corrected and uncorrected deviation, respectively (Table 1).

In this study, we found extensive effusion in 26.3% of TMJs. Retrodiscal edema was seen in one case (1.3%), but no bone marrow edema was detected in any of the patients. Degenerative joint diseases were present in 17.5% of the TMJs, and the mandibular condyle showed a posterior position in the glenoid fossa in 41.3%, an anterior position in 3.8% of the joints. 35% of the joints had anterior DDwR and 26.3% had anterior DDwoR. We observed posterior, medial, and lateral disc displacement in 2.5%, 5%, and 17.5% of TMJs, respectively. Subluxation of the mandibular condyle during the opening was seen in 35%. Abnormal disc morphology was detected in 25%, and thickening of the inferior lateral pterygoid insertion on the disc was present in 35% of the joints. No radiological sign of masseter hypertrophy was evident in the examined cases. The difference between left and right TMJ spaces was less than 1 mm in 82.5%. (Table 2).

**Table 1:** Descriptive statistics of the examined clinical signs

Clinical signs of the examined temporomandibular joints	Frequency (*n* = 80)
TMD pain reported by the patient	44 (55%)
TMJ sounds reported by the patient	56 (70%)
TMJ clicking	41 (51.2%)
Reciprocal click	37 (46.3%)
TMJ crepitation	11 (13.8%)
Condylar hypermobility	33 (41.3%)
Palpation pain in the medial pterygoid muscle	12 (15%)
Palpation pain in the lateral pterygoid muscle	51 (63.7%)
Palpation pain in the masseter muscle	41 (51.2%)
Palpation pain in the temporalis muscle	44 (55%)
Palpation pain in the sternocleidomastoid muscle	8 (10%)
Palpation pain in the lateral TMJ pole	21 (26.3%)
Ear symptom (pain, pop, tinnitus)	23 (28.7%)

Clinical signs of the examined jaws	Frequency (*n* = 40)
Limited mouth opening	2 (5%)
Maximum mouth opening range without pain	50.95 ± 8.13 (mean ± SD)
Maximum mouth opening range with pain	53.43 ± 8.14 (mean ± SD)
Lateral excursion to the right side	9.9 ± 1.91 (mean ± SD)
Lateral excursion to the left side	10.65 ± 2.38 (mean ± SD)
Difference between two-sided lateral excursion < 2 mm	24 (30%)
Difference between two-sided lateral excursion ≥ 2 mm	56 (70%)
Protrusion	8.69 ± 2.3 (mean ± SD)
Corrected deviation	12 (32.5%)
Uncorrected deviation	18 (45%)

**Table 2:** Descriptive statistics of the examined radiologic signs

Radiologic sign	Frequency (*n* = 80)
Effusion	21 (26.3%)
Retrodiscal edema	1 (1.3%)
Bone marrow edema	0 (0%)
Degenerative joint disease	14 (17.5%)
Anteriorly displaced disc with reduction	28 (35%)
Anteriorly displaced disc without reduction	21 (26.3%)
Posteriorly displaced disc	2 (2.5%)
Medially displaced disc	4 (5%)
Laterally displaced disc	14 (17.5%)
Abnormal disc morphology	20 (25%)
Lateral pterygoid muscle insertion thickening	28 (35%)
Masseter muscle hypertrophy	0 (0%)
Mandibular condyle subluxation	28 (35%)
TMJ space width (mm)	2.96 ± 1.03 (mean ± SD)
Difference between two-sided TMJ space < 1 mm	66 (82.5%)
Difference between two-sided TMJ space ≥ 1 mm	14 (17.5%)
Glenoid fossa height (mm)	6.16 ± 1.24 (mean ± SD)
Anteroposterior diameter of glenoid fossa (mm)	17.62 ± 1.63 (mean ± SD)
Mandibular condyle position	
Normal	44 (55%)
Anterior	3 (3.8%)
Posterior	33 (41.3%)

### MRI-based explanation of clinical symptoms

MRI signs indicating pathological changes behind clinical symptoms were analysed using clinical findings as dependent variables in univariate logistic regression models with stepwise selection. MRI factors showing significant correlation with clinical symptoms are presented with statistical parameters of the logistic models in Table 3.

Thirteen regression models were identified, demonstrating significant effects of the independent variables. An odds ratio greater than 1 indicated a positive correlation, while an odds ratio less than 1 indicated a negative relation. A positive association was found in cases of 12 MRI signs and only five MRI signs had a significant negative association with the clinical symptoms.

Palpation pain in the lateral TMJ pole was linked to joint effusion. TMJ crepitation was associated with degenerative joint disease and thickening of the lateral pterygoid insertion. Condylar hypermobility was related to degenerative changes and increased glenoid fossa height. Uncorrected deviation was associated with joint effusion. Palpation pain in the medial pterygoid muscle was linked to effusion and condylar subluxation. Palpation pain in the lateral pterygoid muscle was associated with abnormal disc morphology, TMJ space asymmetry. Masseter muscle pain was associated with degenerative joint disease. In addition, temporalis pain was also linked to anterior disc displacement with reduction and abnormal disc morphology (Table 3).

**Table 3:** Significant factors in the univariate logistic regression models examining clinical signs as dependent variables with p-value and odds ratio

Dependent variable	Independent variables	*P* value	Odds ratio	CI 95%
Palpation pain in the lateral TMJ pole	Effusion	0.049*	2.94	1.01–8.58
TMJ crepitation	Degenerative joint disease	0.015*	5.87	1.24–27.77
TMJ crepitation	Thickening of the insertion of the lateral pterygoid muscle	0.041*	4.90	1.06–22.81
Condylar hypermobility	Degenerative joint disease	0.019*	0.08	0.01–0.70
Condylar hypermobility	Height of glenoid fossa (> 6 mm)	0.02*	4.04	1.28–12.72
Uncorrected deviation	Effusion	0.004*	5.05	1.66–15.25
Palpation pain in the medial pterygoid muscle	Effusion	0.010*	7.32	1.04-51-70
Palpation pain in the medial pterygoid muscle	Subluxation of the mandibular condyle	0.019*	29.15	2.37-357.34
Palpation pain in the lateral pterygoid muscle	Abnormal disc morphology	0.048*	0.87	0.18–0.42
Palpation pain in the lateral pterygoid muscle	Difference between two-sided TMJ space (> 1 mm)	0.022*	0.10	0.02–0.45
Palpation pain in the masseter muscle	Degenerative joint disease	0.011*	7.65	1.58–36.93
Palpation pain in the temporalis muscle	Anteriorly displaced disc with reduction	0.033*	3.64	1.20–10.80
Palpation pain in the temporalis muscle	Abnormal disc morphology	0.012*	0.096	0.02–0.48

* Significant result at significance level of 0.05

### Clinical symptom-based inference of MRI diagnoses

To determine which clinical symptoms can be used to predict MRI signs in order to establish an accurate diagnosis, the radiological sign was used as dependent variable in the logistic regression model, with separate models calculated for each radiological factor. Table 4. contains the regression models with significant independent variables, showing which clinical symptoms have a significant relationship with each radiological factor, i.e. which MR lesion is most likely to appear on the MR image in case of certain clinical signs.

Eleven significant regression models demonstrate how specific clinical signs serve as indicators to infer underlying MRI diagnoses.

Uncorrected deviation and palpation pain in the medial pterygoid muscle point toward the presence of effusion. Condylar hypermobility suggests a lower likelihood of degenerative joint disease, whereas palpation pain in the masseter muscle indicates a higher risk. The presence of palpation pain in the masseter muscle strongly suggests anterior disc displacement without reduction, while condylar hypermobility reduces this possibility. Uncorrected deviation may signal medial disc displacement, TMJ crepitation may indicate thickening of the lateral pterygoid muscle insertion, and palpation pain in the medial pterygoid muscle implies subluxation of the mandibular condyle. Lastly, condylar hypermobility increases the chance of detecting an elevated glenoid fossa on MRI. (Table 4).

**Table 4:** Significant factors in univariate logistic regression models examining radiologic signs as dependent variables with p-value, odds ratio, and AUC value based on the ROC analyses of the regression models

Dependent variable	Independent variables	*P* value	Odds ratio	CI 95%	AUC value
Effusion	Uncorrected deviation	0.012*	7,83	1.97–31.05	0.35*
Effusion	Palpation pain in the medial pterygoid muscle	0.010*	7,20	1.48–34.91	0.37*
Degenerative joint disease	Condylar hypermobility	0.019*	0,08	0.01–0.68	0.29*
Degenerative joint disease	Palpation pain in the masseter muscle	0.011*	7.72	1.52–39.13	0.45*
Anterior disc displacement without reduction	Condylar hypermobility	0.021*	0.22	0.07–0.86	0.64*
Anterior disc displacement without reduction	Palpation pain in the masseter muscle	0.036*	9.33	2.19–39.70	0.65*
Medial disc displacement	Uncorrected deviation	0.042*	11.25	1.09–115.50	0.23
Lateral pterygoid muscle insertion thickening	TMJ crepitation	0.041*	4.00	1.05–15.14	0.41
Mandibular condyle subluxation	Palpation pain in the medial pterygoid muscle	0.019*	4.80	1.29–17.76	0.39
Glenoid fossa height (> 6 mm)	Condylar hypermobility	0.02*	2.98	1.18–7.50	0.63*
Abnormal disc morphology	Palpation pain in the temporalis muscle	0.012	0.25	0.08–0.73	0.33*

* Significant result at significance level of 0.05

ROC curves for each of the predictive logistic models were calculated. (Table 4) Most AUC values indicated inaccurate models, with values below 0.5, suggesting that the model performed worse than random chance. Only anterior disc displacement without reduction with condylar hypermobility and with the pain in the masseter muscle demonstrated statistical significance and a high diagnostic value for the logistic models (AUC = 0.651, SE = 0.067, *P* = 0.024, 95% CI: 0.520 to 0.781; AUC = 0.637, SE = 0.069, *P* = 0.049, 95% CI: 0.501 to 0.773).

## Discussion

This retrospective study investigated the relationships between clinical symptoms of TMD and MRI findings. The aim was to determine the main pathological changes associated with TMD symptoms, and to identify the necessity of MRI examination in clinical practice.

Although numerous studies have investigated the relationship between clinical and MRI findings, most have focused on calculating the diagnostic agreement between clinical examination and MRI results, thereby assessing the reliability of clinical assessment [11, 13, 14, 17, 23] Most of them assessed only anterior disc displacement with and without reduction as final diagnosis and they reported low to moderate reliability of the clinical examinations. Üsümez et al. concluded that MRI may not be necessary in all TMD cases prior to treatment as diagnostic accuracy for identifying disc position was: 83% for normal alignment, 72% for anterior displacement with reduction, and 81% without reduction [17]. Kumar et al. examined both anterior and posterior disc displacement, and they detected the presence or absence of clinical signs and symptoms of temporomandibular disorders with high sensitivity (90%) and specificity (83.3%) [10]. The most recent article comparing the agreement between diagnoses based on DC/TMD clinical examination and MRI, concluded that although DC/TMD clinical examination reliably identified various forms of disc displacement, it demonstrated lower diagnostic accuracy than MRI in detecting degenerative joint disease and condylar subluxation [23].

Some articles analysed the connection based on the frequency of MRI signs in different clinical groups, but these studies mostly investigated anterior disc displacement in relation to internal derangements [9, 12, 16]. Eriksen et al. examined clinical and MRI parameters among TMD group, arthritis group and control group. Both pain on palpation of the TMJs, and limited mouth opening were significantly more prevalent in patient groups compared to controls. However, no statistically significant associations were found between these clinical signs and the MRI parameters investigated [9]. Rudish et al. focused on a group with TMJ pain and concluded that the correlation between clinical pain of the joint and radiologically confirmed TMJ internal derangement or effusion was poor [16]. Mussler et al. examined a group of patients with juvenile idiopathic arthritis and found statistically significant connections between alterations on the condyle and TMJ pain, furthermore, between condyle alterations and mouth opening capacity [12]. 

### MRI-based explanation of clinical symptoms

Only five articles in the literature analysed MRI signs with regression models as potential pathologies behind clinical symptoms [24–28], and our results are partially consistent with these articles. While these studies examined TMJ pain, joint sounds, and limited mouth opening among clinical symptoms, our assessment covered a wider range of clinical findings. In terms of MRI signs, Jeon et al. investigated only three categories of abnormality on MRI: internal derangement, effusion, and disc morphology [24]. Farina et al. extended these factors to include contrast enhancement of joint soft tissues [28], while Matsubara et al. evaluated internal derangement, effusion, condylar morphology and bone marrow signal pattern [26]. Takahara et al. examined anterior disc displacement, osteoarthritis, joint fluid, and bone marrow edema [27]. Chuanje et al. evaluated condylar morphology and condylar position using CBCT, and also assessed disc morphology, disc position, and joint effusion using MR images [25]. While all of these studies focused solely on anterior disc displacement as internal derangement of the TMJ, this recent study examined more types of it, such as posterior, medial and lateral disc displacements. Furthermore, this research contains more abnormalities, including TMJ pain, internal derangement, disc and condylar morphology, effusion, bone marrow pattern, position of the mandibular condyle, muscular pain, and morphology of the fossa were also determined. Another relevant difference between our examination and previous studies is that earlier studies considered types of degenerative disorders such as sclerosis, osteophyte, erosion etc. separately, while we focused only on the presence of obvious degenerative abnormalities, since degenerative signs often appear in combination.

Regarding the results of the aforementioned five articles, Farina et al. confirmed using a univariate regression model that disc displacement, osteoarthrosis, effusion, and synovial tissue enhancement increase the likelihood of painful TMJ [28]. Takahara et al. found a positive association between TMJ pain and anterior DDwoR, severe bony changes, joint fluid, and bone marrow edema [27]. Jeon et al. reported that DDwoR and convex disc configuration decrease the likelihood of TMJ pain, while Matsubara et al. found that high-grade joint effusion was significantly associated with TMJ pain [24, 26]. This latter finding was also confirmed by our present study. In their article, Jeon et al. associated TMJ sound with DDwR, DDwoR, grade 2 and grade 3 joint effusions, and with convex disc configuration. However, they also reported their doubts, as convex disc configuration appeared mainly in DDwoR. Furthermore, they noted that pain and DDwoR showed positive correlations, but pain and convex disc form were negatively associated [24]. Matsubara et al. found a significant positive correlation between TMJ noise and effusion [26]. These results differed from those of our study, in which two factors – degenerative joint disease and lateral pterygoid muscle insertion thickening – were found to increase the likelihood of crepitation. Jeon et al. stated that the likelihood of limited mouth opening increases with a flattened and folded disc configuration and grade 3 joint effusion [24]. Matsubara et al. found that DDwR and DDwoR had a significant effect, but these results could not be confirmed by us [26]. 

Although Chuanje et al. used regression models, they evaluated how certain MRI signs can affect the likelihood of clinically symptomatic TMD [25]. They concluded that, in age groups 19–30 and above 30 years, abnormal condylar morphology, posterior condylar position, DDwR, and DDwoR may increase the likelihood of TMD symptoms.

As our research examined more clinical and radiological variables than previous studies, we found significant connections that had not been published before.

1. Higher glenoid fossa can increase, while degenerative abnormality of the condyle can decrease the likelihood of condylar hypermobility. In most cases, degenerative disorders, including osteophytes, can inhibit movement of the condyle. In the literature osteoarthritic changes were described in relation to jaw limitation, so this may be an explanation for the findings [29].
2. Effusion may lead to uncorrected deviation towards the affected joint. One possible explanation for this association is that the amount of intracapsular fluid and edematous swelling may mechanically restrict the movement of the condyle. Effusion has also been related to palpation pain of the lateral TMJ pole. Therefore, both protective splinting and psychological factors, such as pain-related fear and avoidance learning, may play an important role in this phenomenon [30, 31].
3. Effusion as well as subluxation of the mandibular condyle may be relevant factors in the development of pain in the medial pterygoid muscle. However, there are currently no published data on this connection.
4. Abnormal disc morphology represented a negative association with palpation pain in the lateral pterygoid muscle. Furthermore, a difference of less than 1 mm between the left and right TMJ space increases the likelihood of bilateral pain in the lateral pterygoid muscles, whereas unilateral pain is more common when the difference is more than 1 mm. For these results, we cannot provide a scientific explanation. Although, morphometric variables are not frequently discussed in the context of clinically present pain symptoms in scientific literature, Murphy et al. suggest considering these variables in the context of tissue engineering as treatment option [32].
5. Degenerative malformation showed a positive association with pain in the masseter muscle. Similarly, a compensatory mechanism of the muscles, defence musculaire has been previously described in systemic diseases, e.g. rheumatoid arthritis [33]. Stegenga et al. also described accompanying muscle symptoms in osteoarthrosis of the TMJ [31]. Yap et al. found that pain was associated with early degenerative joint disease. In their study, they found no correlation between the extent of osseous changes and TMD-related pain symptoms, although they did not distinguish between painful sites [34]. Another possible explanation is that both degenerative remodelling and clinical pain in the masseter and temporalis muscles. have a third factor as a cause, namely functional overloading. This factor was not assessed in our retrospective study.
6. Anterior DDwoR as an MRI finding shows a correlation with pain in the temporalis muscle in the causal model, while abnormal disc morphology, such as a flattened disc, decreases the likelihood of temporalis pain. However, these signs and symptoms could also be a result of overloading, which could appear as a causal relationship between anterior DDwoR and temporalis muscle pain in the statistics.

### Clinical symptom-based inference of MRI diagnoses

Regarding the predictive analysis of clinical signs, Üsümez et al. and Yatani et al. assessed the predictability of DDwR and DDwoR using a statistical method other than regression models [17–19]. Üsümez et al. stated that clicking, deviation and pain alone might not always be valid predictors of DDwR. However, when considering the likelihood ratio values of coexisting clicking, deviation and pain in DDwR, the presence of certain clinical signs and symptoms together in the clinical examination may enable a correct diagnosis of the disc–condyle relationship, eliminating the need for MRI confirmation. Moreover, relatively high likelihood ratio values for uncorrected deviation, limited mouth opening, and crepitation suggest that these clinical signs are useful for identifying joints with DDwoR [17]. Yatani et al. examined the diagnostic accuracy of clinical examination for diagnosing anterior disc displacement with reduction. They focused on clicking and click elimination during opening in protrusion and during manipulation according to Dawson. The results of the study also clearly indicate that clicking sounds do not necessarily imply disc reduction or prolapse [18]. According to our analysis, TMJ clicking was not a predictor of radiological anterior DDwR. Yatani et al. also investigated the predictive value of a history of clicking, TMJ pain, limited opening, limited translation, uncorrected deviation, and crepitation in diagnosing anterior DDwoR. Their results suggested that predictability of historical or clinical findings for differentiating anterior DDwoR from other diagnoses was low [19]. 

Only one study was found in the literature that analysed the predictive effect of clinical symptoms using logistic regression to conclude the MRI diagnosis [35]. Emshoff et al. identified four predictive factors of condylar erosion: missing posterior teeth; arthralgia with displacement without reduction (DDwoR) with limited opening MRI finding of DDwoR; and MRI finding of bone marrow edema. However, only the first two could be confirmed by clinical diagnosis [35]. 

Regarding the prediction of MRI signs from clinical symptoms, our study shows that uncorrected deviation, and medial pterygoid muscle pain increase the probability of detecting intra-articular effusion on MRI, with both showing a clinically relevant odds ratio. In case of degenerative joint disease and anteriorly displaced disc without reduction, masseter muscle pain can be a relevant predictive factor for the clinician. However, condylar hypermobility of the TMJ acts as a ‘protective factor’ against both DJD and anterior DDwoR. In theory, medial disc displacement may be predicted from uncorrected deviation. Conversely, while clinicians may be able to conclude to the presence of lateral pterygoid muscle, and mandibular condyle subluxation from TMJ crepitation and pain in the medial pterygoid muscle. In case of higher glenoid fossa, however, condylar hypermobility is only a weak predictive factor.

Considering these recent results, none of the previously published clinical signs have been proven to be strong predictors of an MRI diagnosis. Although our results show some significant effects with high odds ratios, which suggest they could be good predictors, the model evaluation revealed that only one model - in which anterior disc displacement without reduction was associated with condylar hypermobility and palpation pain in the masseter muscle - has acceptable predictive value.

**Fig. 2:**
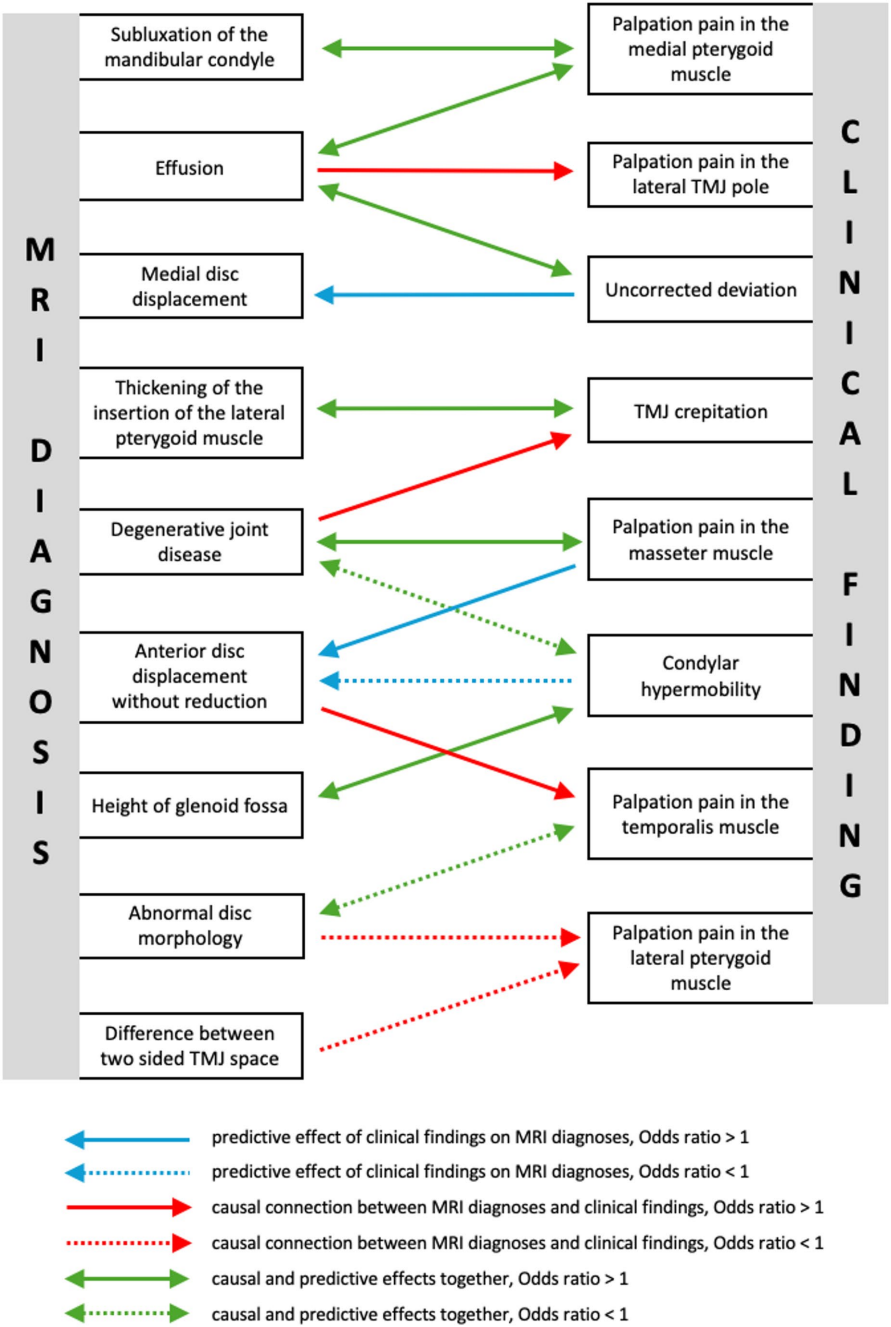
Significant predictive effects of clinical symptoms to MRI diagnosis (blue arrows), pathological connections (i.e. MRI signs) for clinical symptoms (red arrows), and effects of both (green arrows) between MRI signs and clinical symptoms

Based on a comparison of models analysing clinical and MRI signs, eight reciprocal associations were found between clinical symptoms and MRI signs (Fig. 2). Eight correlations were in one direction only. These results suggest that, although the development of a specific clinical sign may be explained by an abnormality seen on MRI, it is not possible to predict this MRI abnormality from a particular clinical symptom. To illustrate this, our results, which correlate with the previously published articles, indicated that effusion may be the most relevant cause of TMJ pain [26–28], however, pain is not a reliable predictive factor for fluid in the joint. Similarly, palpation pain in the lateral pterygoid muscle showed significant associations with MRI abnormalities but cannot be used for predicting any MRI diagnosis. Furthermore, TMJ crepitation may be caused by degenerative abnormalities and lateral pterygoid muscle insertion thickening, but only the latter can be concluded from the crepitation during a clinical examination. (Fig. 2) Another factor complicating prediction is that certain clinical signs are associated with multiple MRI findings. For instance, the presence of uncorrected deviation may suggest both joint effusion and medial disc displacement on MRI. Similarly, condylar hypermobility can indicate degenerative joint disease, anterior DDwoR, and a higher glenoid fossa. Consequently, these characteristics of the connections may hinder accurate prediction. (Fig. 2)

### Limitations

One limitation of this study is its retrospective nature. However, as the same clinician and the same radiologist examined all patients, inaccurate or misinterpreted data collection was avoided. Otherwise, the absence of different examiners also means that intra- and interobserver tests were not performed. Due to retrospective investigation, all examined patients had a clinical TMD symptom, which meant that it was not possible to have a control group in this study. Finally, due to the selection criteria (only fully documented patients without previous orthodontic treatment or treatment for TMD, and no more than one week between the clinical and radiological examinations), the dataset was limited to 80 TMJs.

An exclusion of some originally registered parameters (e.g. pain in the anamnesis) was applied in this study, to gain a less subjective investigation.

## Conclusion

This study confirms that clinical examination alone may be insufficient for accurately diagnosing specific TMJ pathologies. Although some clinical signs show strong associations with MRI findings, only one regression model demonstrated acceptable predictive value.

## Data Availability

The datasets used and/or analysed during the current study are available from the corresponding author on reasonable request.
